# Improved Accuracy of Saxitoxin Measurement Using an Optimized Enzyme-Linked Immunosorbent Assay

**DOI:** 10.3390/toxins11110632

**Published:** 2019-10-31

**Authors:** Jennifer R. McCall, W. Christopher Holland, Devon M. Keeler, D. Ransom Hardison, R. Wayne Litaker

**Affiliations:** 1 Center for Marine Science, University of North Carolina Wilmington, Wilmington, NC 28409, USA; 2 Beaufort Laboratory, National Centers for Coastal Ocean Science, National Ocean Service, National Oceanic and Atmospheric Administration, Beaufort, NC 28516, USA; chris.holland@noaa.gov (W.C.H.); rance.hardison@noaa.gov (D.R.H.); 3 SeaTox Research Inc., Wilmington, NC 28409, USA; dkeeler@seatoxresearch.com; 4 CSS Corporation, Fairfax, VA 22030, USA; wayne.r.litaker@noaa.gov

**Keywords:** saxitoxin, neosaxitoxin, gonyautoxin, paralytic shellfish poisoning, ELISA

## Abstract

Paralytic shellfish poisoning (PSP) is precipitated by a family of toxins produced by harmful algae, which are consumed by filter-feeding and commercially popular shellfish. The toxins, including saxitoxin, neosaxitoxin, and gonyautoxins, accumulate in shellfish and cause intoxication when consumed by humans and animals. Symptoms can range from minor neurological dysfunction to respiratory distress and death. There are over 40 different chemical congeners of saxitoxin and its analogs, many of which are toxic and many of which have low toxicity or are non-toxic. This makes accurate toxicity assessment difficult and complicates decisions regarding whether or not shellfish are safe to consume. In this study, we describe a new antibody-based bioassay that is able to detect toxic congeners (saxitoxin, neosaxitoxin, and gonyautoxins) with little cross-reactivity with the low or non-toxic congeners (decarbamoylated or di-sulfated forms). The anti-saxitoxin antibody used in this assay detects saxitoxin and neosaxitoxin, the two most toxic congers equally well, but not the relatively highly toxic gonyautoxins. By incorporating an incubation step with L-cysteine, it is possible to convert a majority of the gonyautoxins present to saxitoxin and neosaxitoxin, which are readily detected. The assay is, therefore, capable of detecting the most toxic PSP congeners found in commercially relevant shellfish. The assay was validated against samples whose toxicity was determined using standard HPLC methods and yielded a strong linear agreement between the methods, with R2 values of 0.94–0.96. As ELISAs are rapid, inexpensive, and easy-to-use, this new commercially available PSP ELISA represents an advance in technology allowing better safety management of the seafood supply and the ability to screen large numbers of samples that can occur when monitoring is increased substantially in response to toxic bloom events

## 1. Introduction

Harmful algal blooms (HABs) are frequent occurrences in the waters around the world. Historically, HABs have been associated with fish kills and marine mammal mortalities; however, their effects on human health and economic loss due to contamination of seafood are becoming more prevalent. The toxic effects are caused by the toxins produced by various species of marine microalgae and microorganisms, which are consumed by filter feeding organisms such as shellfish. These toxins often vector throughout the food chain, bioaccumulating to varying degrees in shellfish, fish, and birds. Consumption of contaminated seafood can lead to severe and debilitating illness in both humans and marine animals. In the US, the most common illnesses caused by HAB toxin contamination of seafood are from toxins that primarily affect the nervous system and include: ciguatera fish poisoning, paralytic shellfish poisoning, neurotoxic shellfish poisoning, and amnesic shellfish poisoning [1].

**Figure 1:**
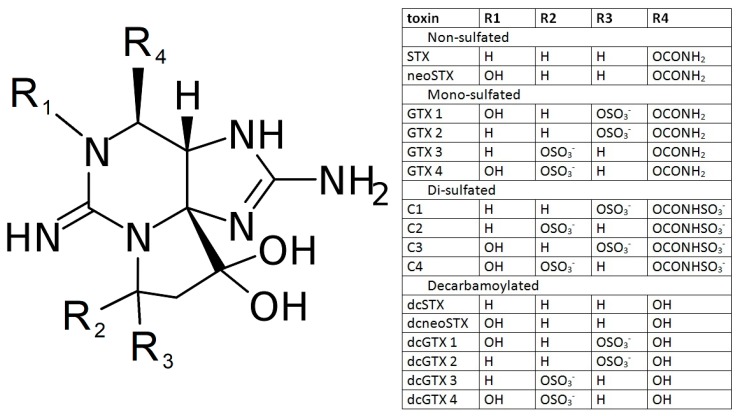
Classes of STX congeners. PSP toxin backbone with various R groups that make toxic and non-toxic congeners of STX.

Paralytic shellfish poisoning (PSP), precipitated by saxitoxin (STX) and its toxic analogs (Figure 1). These toxins activate sodium channels in cell membranes, leading to disruption of neural function. Exposure to sufficiently high levels of PSP toxins can cause respiratory failure and death within hours of exposure if respiratory support is not provided [2,3,4,5]. PSP toxins are produced by various *Alexandrium* spp. and *Pyrodinium* spp. of algae [6], in addition to different species of cyanobacteria. These species principally produce gonyautoxins (GTXs) and sulfocarbomyl (C) toxins, which are then transformed in bivalves to STX and neoSTX, the most toxic congeners. However, these more potent compounds can also be found in various algal strains, comprising up to 20–25% of the total toxin load [7,8,9,10,11,12,13].

PSP toxins can be fatal when consumed, with a rapid onset of symptoms. Some of the milder neurological symptoms include tingling, numbness, ataxia, giddiness, and drowsiness. Respiratory arrest can occur in severe cases within hours of consumption of PSP toxins [6]. In one study, PSP mortality for adults was 7% in cases of severe exposure, but was up to 50% in children, who are more sensitive to the effects of STX [14]. There is no antidote for PSP intoxication, but with supportive therapy (e.g., artificial respiration and CPR), patients can depurate sufficient toxin to allow them to recover [6]. Historically, many of these diseases have been endemic to local areas of HABs, but the increasing globalization of the tourism and fishing industries has led to an increasing number of cases world-wide.

The FDA has established guidelines for the maximum tolerated amounts of marine toxins in seafood of 80 ug/100 g tissue (0.8 ppm) [15]. PSP toxins accumulate and concentrate in the skin, viscera, or meat of various species of filter-feeding shellfish. Since the PSP toxins are heat and acid stable, cooking or otherwise preparing contaminated seafood does not eliminate the risk of poisoning. Therefore, when toxin levels are detected above these limits, entire fishery resources are closed by government regulatory agencies and health departments. However, the decision on what areas to close and for what commercial species is not always clear. For example, resource closures for STXs cannot be based on distribution and concentrations of the toxic algae that produce the toxin because the correlation between algal cell counts and shellfish toxicity is not always clear and is dependent on factor, such as the species of bivalve in question and the suite of saxitoxin congeners being produced by the algae [5,16].

The PSP toxins share a common structure with modifications in functional side groups that governs toxicity (Figure 1). The non-sulfated toxins (STX and neoSTX) are the most toxic to humans, followed by the mono-sulfated toxins GTX 1-4. Relative to saxitoxin, GTX 1 has toxicity of 1.0, GTX 2 of 0.4, GTX 3 of 0.6, and GTX 4 of 0.7 [17]. Di-sulfated (C toxins) and decarbamoylated congeners have little to no toxicity.

Currently, the mouse bioassay is the official regulatory method in most countries for determining PSP toxin levels in seafood samples. Problems associated with the mouse bioassay include: long time requirements (2–24 h to measurable death, and then up to seven days of post-injection observations for no toxicity), high cost ($100 per test), poor specificity (any toxic compound in the sample can kill the mouse), low sample throughput due to labor-intensive requirements, high variability (due to differences in mouse strain, age, and weight), and high animal usage (one animal per test) [16,18]. A receptor-binding assay for PSP toxins has been developed, which measures binding of PSP congeners to sodium channels [19,20]. The advantage of this test is that results are directly correlated with toxicity of the sample, as it detects binding to the sodium channel, which is the mechanism by which PSP toxins cause illness. This assay relies on the use of radioactive compounds produced from the native ligands, specifically tritiated saxitoxin. However, the dependence on radioactivity represents a significant drawback to the widespread use of this assay. Antibody-based tests have the advantage of being user friendly, inexpensive, and reliable, but current commercially available options have been plagued by false positive or negative results of toxicity due to issues with antibody cross-reactivity to extraneous compounds in solution [16].

Ideally, it would be useful to have a quick and cost-effective antibody-based enzyme linked immunosorbent assay (ELISA) method for rapid screening of samples. However, a major impediment has been creating an antibody that can provide selectivity for binding of only toxic PSP congeners, as opposed to the entire suite of toxic and nontoxic congeners. Currently available commercial ELISA kits are unable to detect many of the toxic congeners (e.g., GTXs). To address this issue, we developed an ELISA using an antibody with similar cross-reactivity with the most toxic congeners (STX and neoSTX). In addition, we included a sample incubation with L-cysteine, which converts most of the congeners to STX and neoSTX to allow for accurate toxicity detection in shellfish samples.

## 2. Results

### 2.1. ELISA Optimization

The first step in developing and validating the SeaTox PSP ELISA was to optimize reagent concentration. The optimal antibody concentration was determined by preparing an antibody dilution series and running the ELISA assay in the presence of excess STX-HRP detector to find the greatest dilution that maintained maximal signal without producing false positive results (determined to be 1:700). The optimal STX-HRP detector concentration was then determined by running the ELISA using the optimized amount of antibody and tracer serially diluted in PBS to establish the minimal amount of tracer required to produce the maximal signal and with minimal background. Optimal concentrations were then used for validation of the assay. The dynamic range of the assay included all points of the standard curve from 1.3 ng/mL to 0.16 ng/mL STX. When standard concentrations were run at the lower end of the assay (0.5–0.008 ng/mL), the limit of detection was determined to be 0.03 ng/mL of STX.

Bovine serum albumin (BSA) was used to block the capture antibody plate to determine if that could improve the assay conditions. Reagents were allowed to develop in the absence of any toxin. Maximal OD was 1.23 ± 0.042 without BSA, compared to 1.27 ± 0.076 with BSA blocking (*n* = 3), which was not statistically different. STX-HRP only (no antibody) controls were run to ensure no non-specific binding of the detector occurred. Wells with STX-HRP detector only did not exhibit any detectable color development (data not shown), indicating little to no non-specific binding of the detector. Additionally, two wells of each column received only PBS with no STX (standard or sample) added to serve as controls to establish maximal absorbance and the degree of inter-well variability among replicates. To correct for inter-assay variations due to differences in incubation times or temperatures, all standard or sample absorbance values (B) were divided by average of the absorbance values from the wells where no STX congeners were added (B_0_).

### 2.2. PSP Toxin Antibody Cross-Reactivity

**Figure 2:**
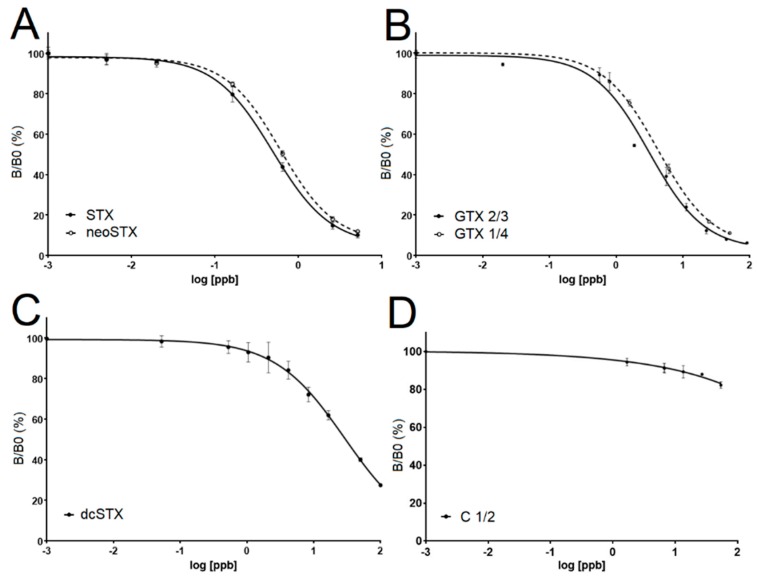
Results of antibody cross-reactivity tests. (**A**) shows saxitoxin (STX) and neosaxitoxin (neoSTX) standard curves generated from the PSP ELISA (*n* = 4). (**B**) shows gonyautoxin 2/3 and 1/4 standard curves generated from the PSP ELISA (*n* = 4). (**C**) shows decarbamoyl STX (dcSTX) standard curve generated from the PSP ELISA (*n* = 6). (**D**) shows C toxin’s (C 1/2) standard curve generated from the PSP ELISA (*I* = 2).

In order to determine the cross-reactivity of the SeaTox antibodies, ELISAs were conducted with pure standards of C 1/2, STX, neoSTX, GTX 2/3, GTX 1/4, and decarbamoyl STX (dcSTX). C 1/2 did not bind sufficiently to the antibody and showed no standard curve (Figure 2D). The maximal percent of B/B_0_ obtained for C 1/2 was still greater than 80% at the highest concentration, and an EC_50_ value could not be obtained. As shown in Figure 2, all other standards were detected by the PSP ELISA but at different sensitivities. EC_50_ values were calculated from the curves from Figure 2 and are shown in Table 1. The corresponding cross-reactivity was then calculated as a percentage of STX detection, as the lowest EC_50_ value obtained (Table 1).

### 2.3. L-Cysteine Conversion Efficiency

**Figure 3:**
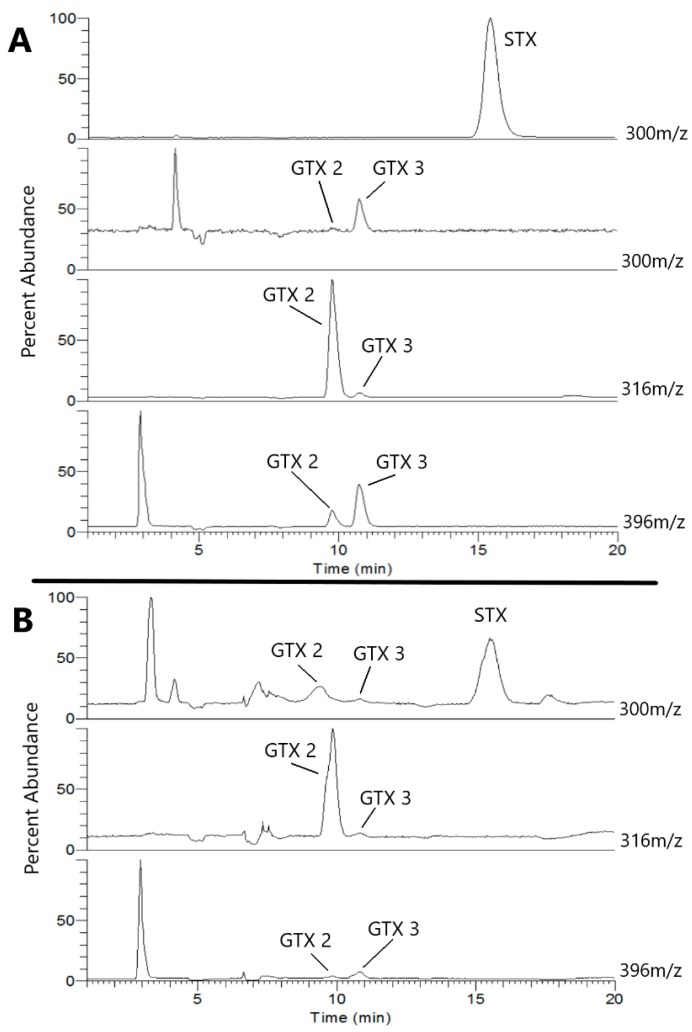
HILIC-MS SIM analyses of separate STX and GTX 2/3 standards are shown in (**A**) for 300, 316, and 396 m/z. (**B**) shows the results of the same analyses of the GTX 2/3 standard following the described cysteine conversion. The appearance of a characteristic STX peak (RT = 15.5 min; 300 m/z) accompanied changes in the GTX peak intensities following the cysteine conversion (B), as compared to the analyses performed on unaltered GTX 2/3 standard (A).

The antibody cross-reactivity showed good detection for the most toxic congeners (i.e., STX and neoSTX), but still showed lowered ability to detect other highly toxic congeners (e.g., GTX 1/4 and GTX 2/3). The addition of L-cysteine has been shown to convert GTXs into STX or neoSTX, which would then be easier to detect with the ELISA [22]. To test this idea, we converted GTX standards with L-cysteine and determined the conversion efficiency with LC-MS (Figure 3).

We then calculated the L-cysteine conversion efficiency of GTX 2 and 3 by determining the percent reduction in abundance of each at 316 m/z (dominant ion of GTX 2) and 396 m/z (dominant ion of GTX 3). As shown in Table 2, GTX 2 was converted at 80% efficiency and GTX 3 was converted at 71% efficiency.

**Figure 4:**
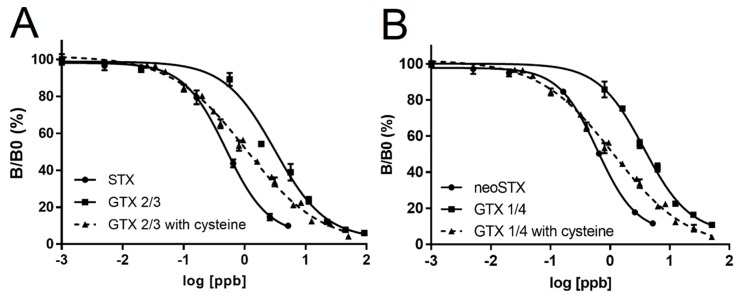
ELISA analysis of the L-cysteine conversion experiments. GTX 1/4 or GTX 2/3 were exposed to an excess of L-cysteine for the conversion to neoSTX or STX, respectively (*n* = 3). (**A**) shows the standard curve results of STX and GTX 2/3 untreated and after L-cysteine treatment, whereas (**B**) shows the standard curve results of neoSTX and GTX 1/4 untreated and after L-cysteine treatment.

To confirm the results of the LC-MS conversion experiments, we ran ELISAs on standard curves for GTX 1/4 and GTX 2/3 after conversion with L-cysteine (Figure 4). For GTX 1/4, the EC_50_ value decreased from 3.694 (Table 1) to 0.858 ± 0.034, which represented a 76.8% reduction. For GTX 2/3, the EC_50_ value decreased from 3.309 (Table 1) to 0.922 ± 0.036, which represented a 72.1% decrease. Both converted EC_50_ values were statistically significantly higher than the EC_50_ values obtained for STX and neoSTX (Table 1), further indicating that the L-cysteine does not completely convert GTXs.

### 2.4. ELISA Validation

After optimizing the ELISA conditions, particularly for detecting toxic GTXs, the final step was to use the SeaTox PSP ELISA to detect PSP toxins in actual shellfish samples. The purpose of this was to demonstrate the ability of the ELISA to detect PSP toxins in a more complicated matrix (i.e., shellfish tissue), as opposed to pure standards. In addition, we sought to determine to what extent the ELISA could reliably estimate toxicity. In order to do this, we compared the results obtained from the ELISA to results obtained from HPLC estimates using previously validated methods [23].

**Figure 5:**
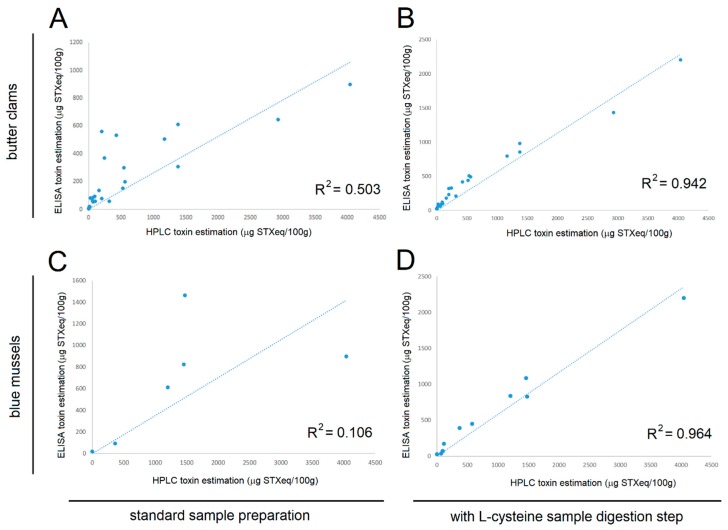
The correlation of HPLC vs ELISA estimated toxicity of shellfish. Samples of butter clams (**A**,**B**) and blue mussels (**C**,**D**) were assayed for toxin content using HPLC and the SeaTox ELISA. The correlation coefficient and slope were calculated. Samples were prepared according to standard methods (**A**,**C**), or prepared with an additional L-cysteine step (**B**,**D**) to convert PSP gonyautoxins congeners to STX and neoSTX for improved detection by the ELISA antibody.

Shellfish samples from butter clams and blue mussels were obtained during a harmful algal bloom. A total of 30 butter clam and 11 blue mussel samples were extracted for toxin testing on both the SeaTox PSP ELISA and HPLC. One dilution of each sample was tested and compared to a standard curve for determination of STX-equivalents. After calculating the STX-equivalents for each individual sample separately on both the ELISA and HPLC, these values were plotted against each other to determine the correlation coefficient for ELISA vs. HPLC (Figure 5).

After shellfish extraction, the amount of PSP toxins in the sample (in STX-equivalents) measured by ELISA showed poor correlation with HPLC (Figure 5A,C), as indicated by *R^2^* coefficient values of 0.5 or less. However, after shellfish extracts were treated with L-cysteine, the STX-equivalence toxicity values determined by ELISA were much more closely correlated with HPLC, yielding R^2^ coefficient values in the range of 0.94–0.96 (Figure 5B,D). As shown in Figure 5, ELISA estimates of toxicity were greatly enhanced after L-cysteine processing of samples. The slope for the HPLC values versus the ELISA values for the butter clams and mussels after incubation with L-cysteine were 0.56 and 0.58, respectively.

## 3. Discussion

Harmful algal blooms produce toxins that can have severe impacts on human and animal health, as well as economic impacts, around the world. These toxins then accumulate in commercially viable seafood, such as shellfish. When contaminated seafood is consumed, it can cause debilitating illness and sometimes death. Seafood fisheries and shellfish beds are often closed to protect human health, but extended closures can be detrimental to the local economy by significantly restricting the activity of fishing communities. It is critical to have inexpensive, reliable, and rapid detection methods to effectively close fisheries when toxic blooms occur, but also to quickly reopen fisheries once the threat has passed.

Rapid assays for PSP toxins are complicated by the large number of STX-backbone congeners that are present in seafood and algal species. There are over 20 different tetrahydropurine compounds in the PSP toxin family [17,18]. Immunoassays are problematic when there are multiple congeners of the same structural backbone, particularly when they vary in toxicity, as in the case of the PSP toxins. Immunoassays are developed using one congener as an antigen, and antibodies raised against that antigen tend to recognize only that one compound, or closely related ones at best. This does not represent a true reflection of toxicity, because non-toxic congeners may bind to the antibody (false positives), or the antibody may fail to detect toxic congeners (false negatives). However, immunoassays such as ELISAs, are easy-to-use and remain the gold standard for rapid detection when more reliable methods, such as HPLC, are too expensive or difficult to use.

In this paper, we report the development and validation of a PSP ELISA using an antibody raised against the neo-STX antigen. This antibody has high cross-reactivity with STX, making it useful for detecting the two most toxic congeners. Native cross-reactivity was low with the next two most toxic congeners, GTX 1/4 and GTX 2/3, which are commonly found in algal and seafood samples. However, the use of a simple L-cysteine conversion step allowed for high fidelity in the detection of these toxins. GTX 1/4 is converted to neo-STX and GTX 2/3 is converted to STX in this reaction [22]. Since the ELISA used for this study was based on an antibody raised against neoSTX, which most other available ELISAs have poor cross reactivity for, the conversion step allowed for a quantitative detection of the most toxic PSP toxins, as indicated by correlation studies using the HPLC standard. We further confirmed the conversion efficiency of GTXs was approximately 70–80%. While not a perfect 100%, these conversion efficiencies still represent a significant improvement in our ability to quantitatively detect the more toxic STX congeners. Furthermore, it is important to note that GTXs are not as toxic as STX or neoSTX, so a complete 100% conversion would result in overestimation of the toxicity of a shellfish sample. There is a risk that incomplete conversion may result in underestimation of toxicity; however, our correlation experiments indicate very tight alignment with HPLC estimates of toxicity. As such, this ELISA coupled with L-cysteine conversion, more reliably estimates toxicity of shellfish samples than other ELISAs and immunoassays (without L-cysteine conversion) that are based on an antibody with little cross reactivity to neoSTX.

The major advantage of this new ELISA is that it is relatively quick, easy, cost effective to perform, and quantitatively estimates the concentrations of the most toxic STX congeners present in a sample. ELISAs are excellent tools to be used as a pre-screening assay for more analytical methods to reduce the time and cost associated with these tests. ELISAs can also be used, if as accurate a reflection of toxicity as this one is, to routinely monitor shellfish beds or aquaculture operations to identify increased risk of toxicity. It also allows rapid screening of samples from different locations during toxic bloom events. The data provided can help managers better protect public health and reduce adverse impacts on local economies. Further studies will need to be completed to gain regulatory authority for routine use of the assay for monitoring toxicity, with this study representing an important first step demonstrating that such an application is possible.

## 4. Materials and Methods

### 4.1. Reagent Acquisition

Antibodies directed against neoSTX and horse radish peroxidase (HRP)-conjugated STX were acquired from SeaTox Research Inc. (Wilmington, NC, USA) for development of this ELISA. NeoSTX was conjugated to glucose oxidase (GOx) (Sigma-Aldrich, St. Louis, MO, USA) using the procedure of Burk et al. [24]. Ten mice were immunized. Serum titers were determined five days after each boost over a period of three months. A fusion was performed on the two mice that showed the greatest immune response. Hybridoma cell lines were prepared and monoclonal antibody production was performed according to the method of Fenderson et al. [25]. The three clones with highest affinity mAbs were selected for further growth and their affinity to different congeners of saxitoxin (STX, dcSTX, neoSTX, GTX 1/4, GTX 2/3, GTX 6) compared. The clone having the greatest overall specificity for the congeners tested was selected for use for assay development in this study. STX was cross-linked to HRP (Sigma-Aldrich, St. Louis, MO, USA) using the procedure of Yoon et al. [26].

### 4.2. Determining Cross-Reactivity of the Antibodies

Saxitoxin standards were purchased from the Certified Reference Materials Program of the Institute for Marine Biosciences, National Research Council Canada (Ottawa, ON, Canada). The certified toxin standards analyzed were as follows: C1/2, dcSTX, GTX2/3, STX, GTX1/4, and neoSTX. To test the cross-reactivity of the antibody to each of these standards, the standards were diluted in 1X PBS (Sigma Aldrich, St. Louis, MO, USA) over a wide range of concentrations. The ELISA assay described below was then run using these samples to empirically determine over what range of concentrations yielded sigmoidal dose response curves so EC_50_ values for each of the standards could be determined. EC_50_ calculations were made Graphpad Prism 6.0 (San Diego, CA, USA) where log of the standard congener concentrations versus the corresponding B/ B_0_ values (see assay procedure below).

### 4.3. ELISA Protocol

A competitive ELISA was developed as follows. All reagents and standards/samples were brought to room temperature prior to use. 50 µL of anti-PSP antibody solution was pipetted into each well of a 96-well microplate pre-coated with goat-anti mouse antibody (Thermo Fisher Scientific, Waltham, MA, USA). Next, 50 µL of standard or sample were added to wells in duplicate. The reactions were allowed to incubate for 30 min to allow the anti-PSP antibody to bind to the goat anti-mouse antibodies bound to the plate and to allow for any sample to also be captured by the bound anti-PSP antibody. Plates were placed on an MSI S1 Minishaker (IKA Works, Inc. Wilmington, NC, USA) set at 800 rpm to ensure the reagents in the microtiter wells mixed thoroughly. After 30 min, 50 µL of STX-HRP detector was added to each well and the assay plate was returned to the Minishaker, allowing the STX-HRP to bind to any anti-PSP antibody not bound to the toxin. Following incubation, plates were washed three times with 200 µL of wash concentrate (PBS buffer with 1% tween) on an ELx 50 plate washer (Biotek, Winooski, VT, USA). Next, 100 µL of TMB substrate solution (Thermo Scientific, Rockford, IL USA) was added to each well and shaken for at least 5 min to ensure complete color development. Following development, 100 µL of acid stop solution was added to each well. Final absorbance of the standard curve and samples were measured at 450 nm on a ClarioStar fluorometer (BMG Labtech, Cary, NC, USA).

### 4.4. Tissue Extraction and Preparation

The Association of Analytical Chemists (AOAC) standard method developed by Lawrence et al. [23] was used to extract saxitoxins from tissues of butter clam and blue mussels collected in Alaska. Briefly, 5 g of tissue was homogenized in a 50 mL tube, and then extracted in 3 mL of 1% acetic acid. Samples were boiled for 5 min with loose caps, allowed to cool to room temperature, vortexed for 30 s, and centrifuged at 4700 rpm for 10 min. The remaining supernatant was poured into a graduated 15 mL conical tube (BD Falcon, New York, NY, USA). Next, 3 mL of 1% acetic acid was added to the 50 mL tube containing the residue as a wash and vortexed for 30 s again. The 50 mL tube was again centrifuged and the supernatant was added to the previous 3 mL in the 15 mL tube. If samples did not clear despite centrifugation steps, those samples were transferred to a syringe, and then passed through a 0.45 mm Millex HA syringe filter (Millipore, Billerica, MA, USA) which cleared the sample. The combined extracted supernatant was then brought to 10 mL using Milli-Q DI water and stored at −20 °C until ready for analysis.

To enhance detection in the assay, L-cysteine was added to some samples to convert PSP congeners to STX or neoSTX. For each sample, two wells were run with samples treated with L-cysteine and two were run without the 70 °C L-cysteine treatment. The conversion of congeners to STX and neoSTX used the procedure developed by Turnbull et al. [22]. Briefly, 0.075 g of L-cysteine (Sigma Aldrich, St. Louis, MO, USA, catalog #w326305) was weighed and added to a 2 mL vial. Next, 300 µL of extracted sample was added to the vial, vortexed, and incubated in a water bath for 30 min at 70 °C. The mixture was cooled on ice for 5 min and homogenized. After incubation, standards or samples were serially diluted, the ELISA protocol was performed as above, and EC_50_ values were calculated for the standard curves to then be compared with HPLC results. Testing of the shellfish ELISA plate also included a five-point STX standard curve (1.3, 0.64, 0.32, 0.16, and 0 ppb), where each concentration was run in duplicate.

The linear portion of the standard curve where samples are quantifiable falls between B/B_0_ values of ~20% and 80%. Outside this range, STX was detectible but not accurately quantified. Samples with a B/B_0_ less than 20% can be diluted so they fall in the 20–80% range. To determine the B/B_0_ level where STX was no longer detectable, we averaged all the B/B_0_ values from the controls (no STX added). Once the control standard deviation was known, we selected the conservative B/B_0_ value of 90% to represent the lower limit of detection for the assay. This value fell more than two standard deviations below that determined from the control standard deviation, greatly reducing the chances of false positive readings. Sample B/B_0_ values falling between 80% and 90% were classified as containing detectible, but not quantifiable (DBNQ), levels of STX.

### 4.5. HPLC Analysis

The butter clam and blue mussel tissue samples were tested for STX concentration using HPLC as previously described [23]. The total toxicity of the sample was expressed as STX-equivalents using a weighting system based on the relative toxicity of the congener (Figure 1). The extraction protocol used for HPLC was identical to the one used to prepare the ELISA samples with the exception of a subsequent SPE purification step in the HPLC protocol. Those homogenates were stored at −80 °C and thawed at room temperature under a culture hood before being mixed and analyzed using the SeaTox ELISA as described above.

### 4.6. L-Cysteine Conversion LC-MS Detection

Mass spectral experiments were performed using a Thermo Scientific Q Exactive Plus hybrid quadrupole-Orbitrap mass spectrometer equipped with a Thermo Scientific Vanquish UHPLC system (Thermo Fisher Scientific, Waltham, MA, USA). HILIC-MS analyses of PSP toxins were carried out with a 5 µm TSK-gel Amide-80 column (250 mm × 4.6 mm i.d.) (Tosoh Bioscience LLC, Montgomeryville, PA, USA). Mobile phases consisted of (A) 100% DI MilliQ filtered water and (B) 95% acetonitrile to 5% water (*v/v*), both containing 0.1% formic acid and pH adjusted to 5.5 with ammonium formate (VWR International LLC., Radnor, PA, USA). LC conditions consisted of 65% (B) at 0.8 mL/min with column temperature at 25 °C and 5 µL injection volumes. Targeted SIM analyses were performed in positive ionization mode with targeted m/z including 300, 282, 396, 316, and 298 m/z. Each NRC PSP toxin standard was analyzed before 50 µL were combined with 12 mg of cysteine, incubated in a water bath at 70 °C for 30 min, and then analyzed again. Conversion efficiency was calculated per toxin as the percent decrease in intensity of the starting toxin’s prominent ion following L-cysteine processing.

## Figures and Tables

**Table 1:** EC_50_ values generated from ELISA curves, with corresponding percentage of antibody cross-reactivity. EC_50_ values were determined from individual curves (*n* = 4 for STX, neoSTX, GTXs and *n* = 6 for dcSTX), and results are presented as average ppb ± standard deviation. The cross-reactivity of another PSP ELISA from Abraxis, as reported in their kit instructions, is provided for comparison [21].

PSP Congener	EC_50_ (in ppb)	SeaTox Cross-Reactivity	Abraxis Cross-Reactivity
STX	0.489 ± 0.031	100%	100%
neoSTX	0.608 ± 0.032	80.4%	1.3%
GTX 1/4	3.694 ± 0.352	13.2%	0.2%
GTX 2/3	3.309 ± 1.144	14.8%	23%
dcSTX	49.15 ± 43.58	9.9%	29%

**Table 2:** LC-MS detection for conversion experiments of GTX2/3 to STX. Pure standard STX and GTX 2/3 were assayed by LC-MS before and after an L-cysteine conversion experiment. Conversion efficiency was determined by calculating intensities of the dominant ion (bold). Signature STX masses and retention time were observed following cysteine conversion of the pure GTX congeners, where they had previously not been present.

PSP Congener	RT (min)	M+H	Fragment Ion	Reduction in Abundance
STX	15.5	**300**	282	
GTX 2	9.8	396	**316** (298)	
GTX 3	10.8	**396**	316 (298)	
GTX 2 + L-cysteine	9.8	396	**316**	80%
GTX 3 + L-cysteine	10.8	**396**	316	71%
